# Medical Specialties

**Published:** 1865-08

**Authors:** 


### Editorial.

### MEDICAL SPECIALTIES.

We copy from the Chicago Medical Journal the very excellent address of Prof. Brainard, before the American Dental Association, upon Medical Specialties. A better presentation of the subject we have never seen. It is a grand refutation of all the objections we have ever heard urged, against a division of medical practice. It is a presentation of the subject that would be exceedingly interesting to any and all studying and practicing any particular branch of medicine. Those who sneer at specialists, and imagine that they can attain perfection in every particular, are handled without gloves. It should be in the hands of every one engaged in every department of the healing art; indeed, it would be exceedingly valuable for distribution to many people outside of the profession.

So fully impressed have we been with the value of the address, and its influence for good, that we have had it stereotyped, and shall issue a large edition in pamphlet form, for those who may choose to order them, for gratuitous distribution. To all such we will furnish them at the cost of paper and printing, which will probably be from $2 00 to $2 50 per hundred. Any one desiring them will please notify us as early as possible.

We have done this because of the universal desire we heard expressed by members of the Association for the general distribution of this address.

